# In silico analysis of promoter region and regulatory elements of mitogenome co-expressed *trn* gene clusters encoding for bio-pesticide in entomopathogenic fungus, *Metarhizium anisopliae*: strain ME1

**DOI:** 10.1186/s43141-021-00191-6

**Published:** 2021-06-22

**Authors:** Getachew Bantihun, Mulugeta Kebede

**Affiliations:** Department of Applied Biology, School of Applied Natural Science, Adama Science and Technology University, P.O. Box 1888, Adama, Ethiopia

**Keywords:** CpG islands, Entomopathogen, Motif, promoter, Transcription factor, Transcription start site

## Abstract

**Background:**

Pest control strategies almost entirely rely on chemical insecticides, which cause environmental problems such as biosphere deterioration and emergence of resistant pests. Bio-pesticide is an alternative approach, which uses organisms such as entomopathogenic fungi, *Metarhizium anisopliae*, to control pests. Screening such potential organism at a molecular level and understanding their gene regulation mechanism is an important approach to reduce emergence of pesticide resistance and worsening of the biosphere. Understanding promoter regions which play a pivotal role in gene regulation is crucial. In particular, identification of the promoter regions in *M. anisopliae* Strain ME1 remains poorly understood. To our knowledge, the mitogenome *trn* gene clusters of *M. anisopliae* Strain ME1 were not characterized. Here, we used machine learning approach to identify and characterize the promoter regions, regulatory elements, and CpG island densities of 15 protein coding genes of entomopathogenic fungi, *M. anisolpliae* Strain ME1.

**Results:**

The current analysis revealed multiple transcription start sites (TSS) for all utilized sequences, except for promoter region genes of Pro-cob and Pro-nad5. With reference to the start codon (ATG), 85.3% of TSS was located above – 500 bp. Based on the standard predictive score at cut off value of 0.8^a^, the current study revealed 54.7% of predictive score greater than or equal from 0.9 promoter prediction score. Expectation maximization algorithm output identified five candidate motifs. Nonetheless, of all candidate motifs, MtrnI was revealed as the common promoter region motif with a value of 76.9% both in terms of size of binding sites and with an *E* value of 9.1E−054. Accordingly, we perceived that MtrnI serve as the binding site for tryptophan cluster with *P* value 0.0044 and C4 type zinc fingers functions as the binding site to regulate gene expression of *M. anisopliae* Strain ME1. The analysis revealed that mitogenome *trn* gene clusters of *M. anisopliae* Strain ME1 showed homologues evolutionary ancestor supported with a bootstrap value of 100%.

**Conclusion:**

Identified common candidate motifs and binding transcription factors through in silico approach are likely expected to contribute for better understanding of gene expression and strain improvement of *M. anisopliae* Strain ME1 for its bio-pesticides role.

## Background

Existing control strategies of pests rely almost entirely on chemical insecticides where its overuse causes environmental problems related to pesticide pollution [1]. These problems are complex due to the high load of recalcitrant substances that are not ecofriendly [2]. As chemical pesticides deteriorate the biosphere and cause emergence of pesticide resistant, pathogens have created opportunities to replace them as environmentally friendly bio-pesticides [3, 4].

Among bio-pesticides, the pathogenic *Metrahzium anisopliae* which belongs to ascomycete family is the most important and promising candidate in the natural regulation of insect populations [5]. According to Gao et al. [6] the product units of *M. anisopliae* enable the infection of target insects through mechanisms of adhesion and penetration of the host cuticle. Besides to this, studies have been conducted to understand the molecular level infection pathways of insects by examining *M. anisopliae* species, for example: laboratory tests using *M. anisopliae* against ticks [7], controlling *rice leaf roller* [8], and mortality of migratory locust [9]. There is evolving evidence that insect pathogenic fungi are unique in being able to infect across the insect cuticle [10] and attack the insect by invading cuticle layer [11, 12]. In contrast, other organisms like viruses and bacteria attack their host after ingestion [5].

Promoters are key elements that belong to non-coding regions [13] located adjacently upstream of transcription start sites and control the activation or repression of the genes [14]. According to Won et al. [15], predicting the promoter region or the transcription start site (TSS) is important as it allows one to study the functional roles of genes through identification of transcription factors. Conserved DNA sequences or consensus regions which are involved in a gene regulation are stored with the region of motif. Finding those conserved regions is a systematic approach for better understanding of regulatory mechanisms [16]. Identification and characterization of promoter regions of *M. anisopliae* will allow earning potential knowledge of gene expression profiles and regulatory mechanisms of gene regulation. This knowledge is important for rapid identification of genes encoding biologically active molecules and eventually to suggest strain improvement techniques [17–19]. Although entomopathogenic role of *M. anisopliae* Strain ME against insects was discussed, there is no report yet about regulatory elements and their respective binding sites involved in gene expression of mitogenome *trn* gene clusters. Therefore, this study was intended to analyze promoter regions and possible transcription factors with the aid of TSS, such as regulatory elements and sequence motifs of entomopathogenic fungi *M. anisopliae* Strain ME. The knowledge will contribute to the strain screening and improvement approaches of *M. anisopliae* Strain ME1 for the purpose of pest control.

## Methods

### Determination of transcription start site and promoter regions

Mitochondrial genome (mitogenome) sequence of *M. anisoplia*e Strain ME1 genes responsible for hydrolyzing insect cuticle were searched from NCBI genome browser (https: //www. ncbi.nlm.nih.gov/gene). Following checking search results from the sequence database, 15 protein coding sequences were considered for the current analysis. Availability of start codons were considered as a part of functional gene for further analysis. The region of transcription start site (TSS) was determined by extending sequences from the genomic coordinate region. Query sequences in FASTA format was used as an input data. The prepared sequence was taken to Neural Network Promoter Prediction (NNPP version 2.2.) tool to obtain 1 kb sequence upstream from a TSS region, assuming this region would contain the potential core promoter. The NNPP version 2.2 tool set was used with the minimum standard predictive promoter score with a default cut off value 0.8 for eukaryotes intended to eliminate zero counts by 80% from the query sequence before transformation [20]. Based on the output of NNPP, promoter prediction sequence regions for those containing more than one TSS, the highest prediction score was considered for trustable and accuracy cut off values. The remaining TSS regions were just utilized for simple comparative analysis.

### Analysis of sequence motif and transcription factors

Upstream 1 kb sequence region from a TSS as a rule of thumb by the help of NNPP considered as a promoter region of *M. anisopliae* Strain ME1 was imported and analyzed using expectation maximization algorithm (MEME) 5.1.1 version via web server (http://meme-suite.org/tools/meme). Through this technique, the occurrence of the common motifs that serve as the binding sites for the transcription factors expected to regulate the expression of mitogenome *trn* gene clusters was determined. MEME Suite was used for performing motif discovery, motif alignment, motif location and distribution, binding site enrichment analysis, motif scanning, and motif–motif comparisons [21]. Before starting search for typed sequences, basic search parameters for motif distribution menu were set, including motif site distribution, option zero, or one occurrence per sequence or model type zoops. Whereas, the number of motifs and the remaining motif width were kept as a default. Once the MEME search was completed, the search result page was linked to the MEME output in HTML format. Following collection of common motif, analysis for a better characterization of those motifs was done through button on the MEME HTML output. It was also a fundamental initial point to look for the expectation value (e value) even though not always the best hit comes in first line with a principle of the smallest the e value, the better the match. Accordingly, the first discovered motif was submitted for further analysis. The MEME output for each motif was forwarded with a button for submitting that motif directly to TOMTOM [22]. The query sequence data *M. anisopliae* Strain ME1 genes were compared with known motifs from target database through TOMTOM, web-based searching motif comparison programs. Prediction of matching transcription factors with the query motifs, alignment profile, and frequency matrix of sequence logos were evaluated by the help of TOMTOM [23]. Position-based motifs, binding domains, and its peaks were predicted by the help of Regulatory Sequence Analysis Tool. For this analysis, JASPAR core and uniprobe mouse were used as reference database binding motifs.

### Gene ontology (GO) analysis for sequence motifs and enrichment analysis

Functional role of a retrieved sequence motif was determined by analyzing GOMO (Gene Ontology for candidate Motifs) version 5.0.1 searches. Furthermore, GOMO or GO was used to compare matching of upstream regions of any sequences with discovered motifs by MEME. A computational method Gene Set Enrichment Analysis (GSEA) genemani (*https://genemania.org/search/homo-sapiens/NDUFS5**)* was utilized to find out the associations of differentially expressed genes with a certain biological process [19, 24]. With the help of this tool, gene pathways and expression correlations were compared with database reference genes.

### Analysis of CpG islands

A 2-kb length of FASTA format query sequence upstream of the start codon was prepared for all 15 protein coding sequences of *M. anisopliae* Strain ME1. The regulatory region, CpG islands which represent regions of a sequence was studied with two algorithms. The first algorithm was the offline tool CLC Genomics Workbench version 3.6.5 (http://clcbio.com, CLC Bio, Aarhus, Denmark) used for searching the restriction enzyme *MspI* cutting sites (with fragment sizes between 40 and 220 bp parameters), and the second tool was Takai and Jones [25] algorithm with search criteria in GC content ≥ 55%, Observed CpG/Expected CpG ratio ≥ 0.65, and length ≥ 500 bp.

### Phylogenetic tree construction

The phylogenetic tree was constructed using MEGA X version 10.18 platform through the Neighbor-Joining method [26, 27] using aligned protein sequences of fungal mitogenomes. The phylogenetic relationship for *M. anisopliae* Strain ME1 (15 conserved protein coding genes) was inferred with significant 20 aligned sequences of fungi strains. The bootstrap test of phylogeny was performed with 1000 repetitions. Bootstrap test was used for evaluation of the reliability of each specific clade in the tree. Some parameters like statistical method, test phylogeny, and number of bootstraps in phylogenetic menu were modified just for analysis preferences so as to obtain stable estimates of reliable tree.

## Results

### Identification of transcription start site

To characterize upstream of the promoter region, each gene was analyzed with regions of 1 kb upstream of transcriptional start site, indicating that functional elements of the core promoter may lie up within this region. Except promoter regions, pro-cob, and pro-nad5 with single TSS, all others have multiple TSS (Table 1). However, for those promoter regions with more than one TSS, high score value was considered to determine core promoter regions, which were shown in bold. Based on distance from the start codon to each TSS location, the current study revealed that 14% of the TSS was located below – 500 bp and subsequently 85.3% of the TSS was situated above – 500 bp. When predictive score at cut off value of 0.8^a^ was considered, 54.7% of predictive score were greater than or equal for 0.9 values.

**Table 1:** Predictive cutoff score and number of transcription start site for the corresponding genes

Gene ID	Corresponding promoter region name	No. of TSS identified	Predictive score at cutoff value 0.8^a^	Distance from start codon (ATG)
4097430	Pro-atp6	3	0.82, 0.82, **0.95**	– 1450, – 453, – 445
4097429	Pro-atp8	3	0.82, 0.82, **0.95**	– 1195, – 198, – 190
4097423	Pro-atp9	6	0.89,0.88, 0.91, **0.96**, 0.92, 0.89	– 1859, – 1804, – 1705, – 1461, – 965, – 576
4097424	Pro-cob	1	0.84	– 2290
4097425	Pro-cox1	3	0.84, 0.85, **0.87**	– 1231, – 1159, – 431
4097419	Pro-cox2	6	0.89, 0.88, 0.91, **0.96**, 0.92, 0.89	– 2192, – 2136, – 2038, – 1794, – 1,298, – 909
4097431	Pro-cox3	9	0.97, 0.85, 0.92, 0.95, **0.99**, 0.97, 0.89, 0.92, 0.92	– 2425, – 2242, – 1804, – 1598, – 1421, – 1280, – 1198, – 1,045, – 340
4097426	Pro-nad1	5	0.87, 0.81, **0.99**, 0.91, 0.92	– 2276, – 1667, – 1476, – 829, – 11
4097421	Pro-nad2	5	0.96, **0.98**, 0.96, 0.82, 0.80	– 2,949, – 1916, – 1517, – 1405, – 1 230
4097422	Pro-nad3	6	0.89, 0.88, 0.91, **0.96**, 0.92, 0.89	– 1310, – 1254, – 1156, – 912, – 416, – 27
4097428	Pro-nad4	5	0.87, 0.81, **0.99**, 0.91, 0.94	– 3494, – 2885, – 2694, – 2047, – 1227
4097420	Pro-nad4L	6	0.89, 0.88, 0.91, **0.96**, 0.92, 0.89	– 3149, – 3093, 2995, – 2751, – 2255, – 1866
4097417	Pro-nad5	1	0.84	– 95
4097432	Pro-nad6	12	0.97, 0.85, 0.92, 0.95, **0.99**, 0.97, 0.89, 0.92, 0.92, 0.92, 0.83, 0.87	– 3420, – 3237, – 2799, – 2593, – 2416, – 2275, – 2193, – 2,040, – 1,335, – 969, -717,-56
4097418	Pro-rp3	4	0.93, 0.87, 0.90, **0.95**	– 1898, 1725, – 1301, – 983

^a^NNPP version 2.2 tool set was used with the minimum standard predictive promoter score with a default cut off value 0.8

### Determination of common candidate motifs and TFs

Five candidate motifs were discovered by MEME algorithm (Table 2). Both Mtrn I and Mtrn III candidate motifs were equally shared binding site distributions with 76.9%; however, there was variation based on statistical expectation value (e value).

**Table 2:** Identified common candidate motifs of mitogenome promoter regions of *M. anisopliae*

Discovered candidate motifs	No. of *trn* promoters for each of the motifs in %	E value	Motif width	Number of binding sites
Mtrn I	10 (76.9)	9.1E−054	50	10
Mtrn II	9 (69.2)	2.0E−048	50	9
Mtrn III	10 (76.9)	4.6E−047	50	10
Mtrn IV	6 (46.2)	1.0E−038	50	6
Mtrn V	8 (61.5)	1.6E−039	50	8

**Fig. 1:**
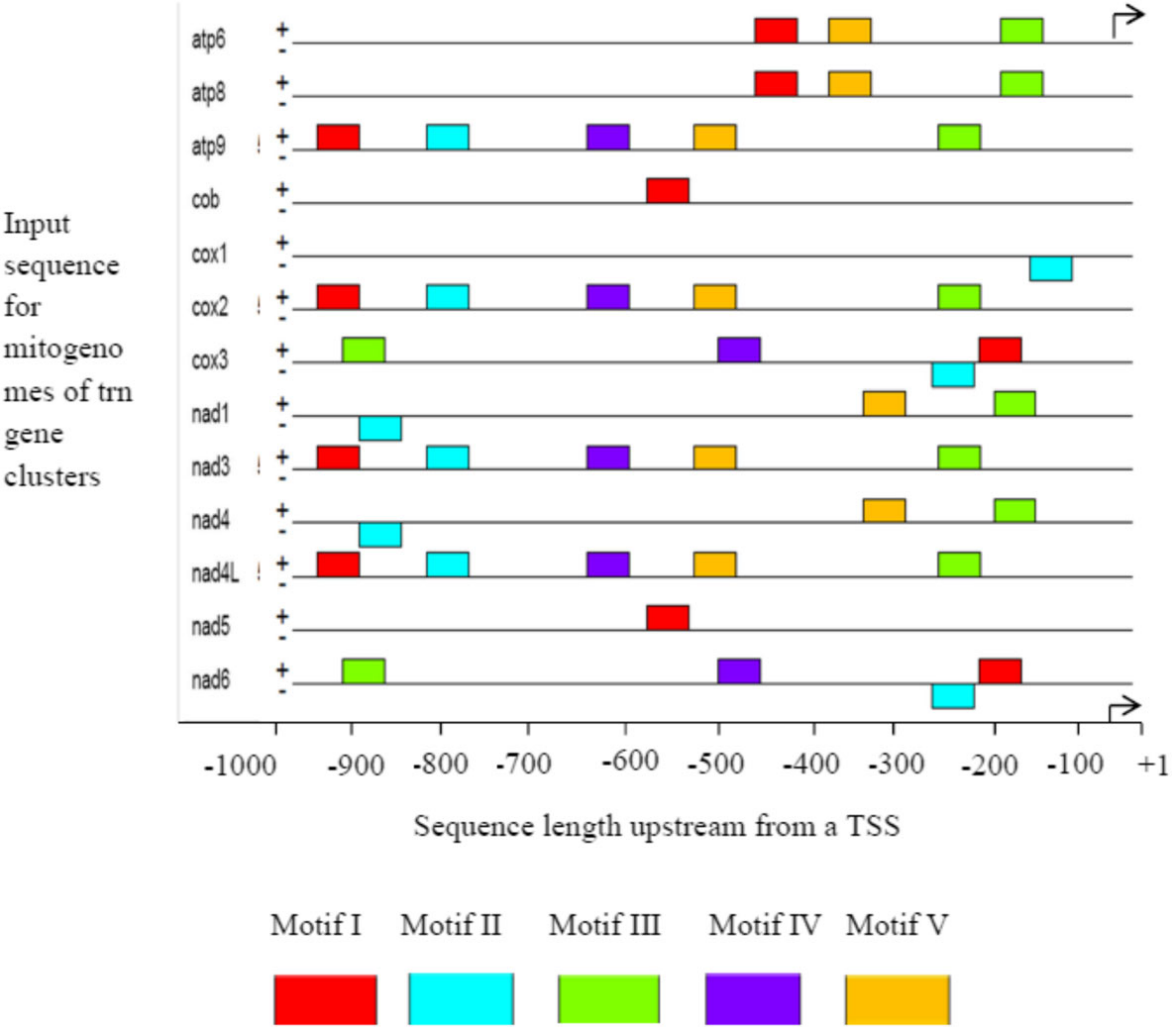
Block diagrams show distribution and location of input promoter sequence of *trn* gene candidate motifs

Since transcription factors often work in concert with each other, it is good to identify the distribution of all the motifs recognized by MEME. Accordingly, MEME generated thirteen candidate motifs, which were distributed from position of TSS (+ 1) to the upstream of − 1 kb (Fig. 1).

**Fig. 2:**
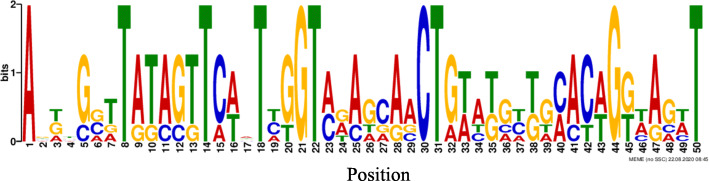
Sequence logos for the identified common Mtrn I motif with different information contents

In the current analysis, candidate Motif I and Motif III were distributed in the positive strand region with the highest number of binding sites. More distribution and location of the majority candidate motifs (69.8%) were observed in between – 600 bp to – 200 bp with the reference to the transcription start site region. To address the information content, MEME created sequence logos for the common motif Mtrn I, which resulted in different characters of motif alignment columns, where the height of the letter represents how frequently that nucleotide is expected to be observed in that particular position (Fig. 2). This motif was revealed as a common promoter sequence gene with 76.9% binding site composition for gene regulatory elements or TFs involved in the regulation of gene expressions.

Common motif Mtrn I was compared with the database registered publically available matching motifs; accordingly, four matching motifs with the query motifs were obtained (Table 3). Accordingly, this study revealed that the common motif Mtrn I bear a resemblance with transcription factor families including Myb domain factors, RXR-related receptor, and thyroid hormone receptor-related factors. However, statistically the candidate transcription factor, tryptophan cluster factors showed the highest binding ability with the Mtrn I common motif.

**Table 3:** List of matching candidate transcription factors (TFs) which could bind to common Mtrn I motif

Gene ID	Gene Name	Species	TF Family	Candidate TF	Statistical significance ^a^
MA0100.3	MYB	*Mus musculus*	Myb/SANT domain factors	Tryptophan cluster factors	**0.00440**
MA0114.3	Hnf4a	*Mus musculus*	RXR-related receptor (NR2)	Nuclear receptors with C4 zinc fingers	**0.00465**
MA0857.1	Rarb	*Mus musculus*	Thyroid hormone receptor-related factors (NR1)	Nuclear receptor with C4 zinc fingers	**0.00524**
MA0859.1	Rarg	*Mus musculus*	Thyroid hormone-receptor-related factors (NR1)	Nuclear receptors with C4 zinc fingers	**0.00556**

Transcription activator candidate tryptophan cluster factors play a pivotal role in the control of proliferation as it was revealed in the UniProt database. Similarly, contribution in gene expression activation and growth function by both retinoic acid receptor beta (rarb) and retinoic acid receptor gamma (rarg) genes were revealed in this study.

### Gene ontology (GO) analysis for the identified sequence Mtrn I motif

**Fig. 3:**
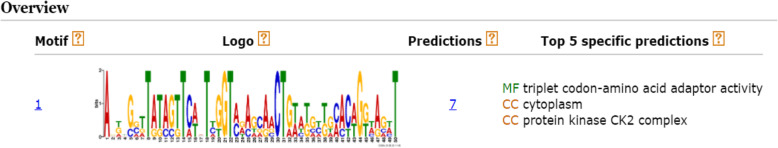
Logo sequence for investigated common Mtrn I motif

The present study identified GO terms for the identified candidate common motif Mtrn I mitogenome *trn* gene promoter sequence of *M. anisopliae* Stain ME1 (Fig. 3). Common motif Mtrn I searched through MEME suite has resulted in domains with defined molecular functions and biological process.

Interestingly, gene product designated as CG33237 has described functional associations with the query candidate motif in relations to the molecular role for protein kinase regulator activity and biologically involved during regulation of protein serine or also in phosphatase activity (Table 4).

**Table 4:** GO terms for motif Mtrn I

Genes	GO molecular function	GO biological process
CG33237	Protein kinase regulator activity	Regulation of protein serine/threonine phosphatase activity
mitochondrial Cytochrome b/Cyt-b	Ubiquinol-cytochrome c-reeducates activity	Spermatogenesis mitochondrial, ubiquinol to cytochrome c
tRNA_gene/Arg-ACG-1-2	CGU codon-amino acid adaptor activity	Translation

### Analysis of promoter regions of co-expressed genes

**Fig. 4:**
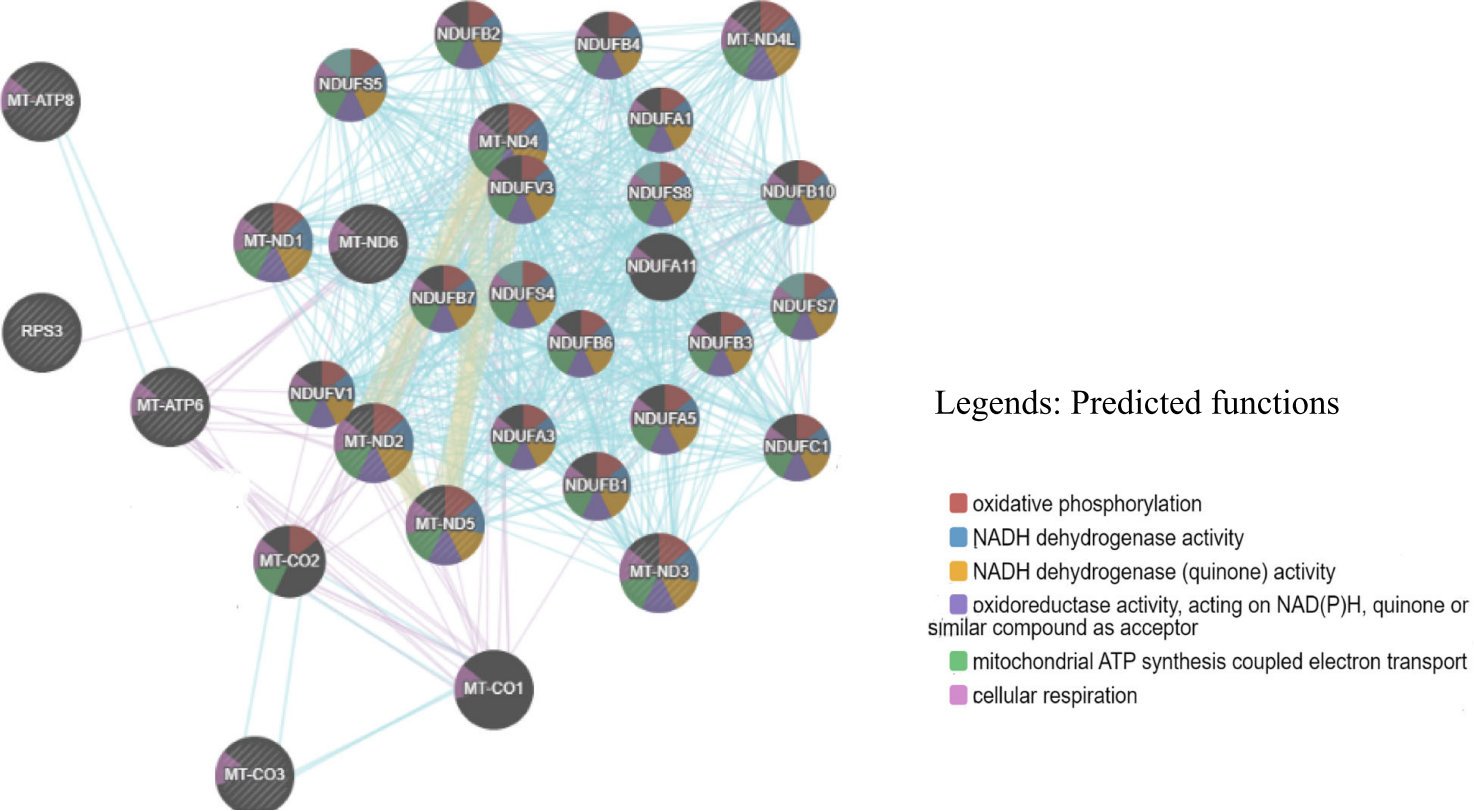
Predicted functional interactions of mitogenome *trn* promoter region gene clusters by GSEA pathway

Gene set enrichment analysis (GSEA) comparison revealed genetic interactions for mitochondrial-encoded gene promoter regions with publically available database gene members. Promoter region gene sequence for ATP synthase membrane subunit 8 showed significant relations with only atp6 and its relation were for 0.50%. Among all analyzed promoter region genes, only nad1 showed interactions with N-formylmethionine representing drug interactions. Overall, when the networking among the promoter region genes against predicted functional genes has been considered (Fig. 4), the genes used in the current study shown 52.63% of co-expression activity with other genes, shared protein domains with 29.02%, pathways (13.83%) and drug interaction (4.52%).

### Description and analysis of motifs by using RSAT

**Fig. 5:**
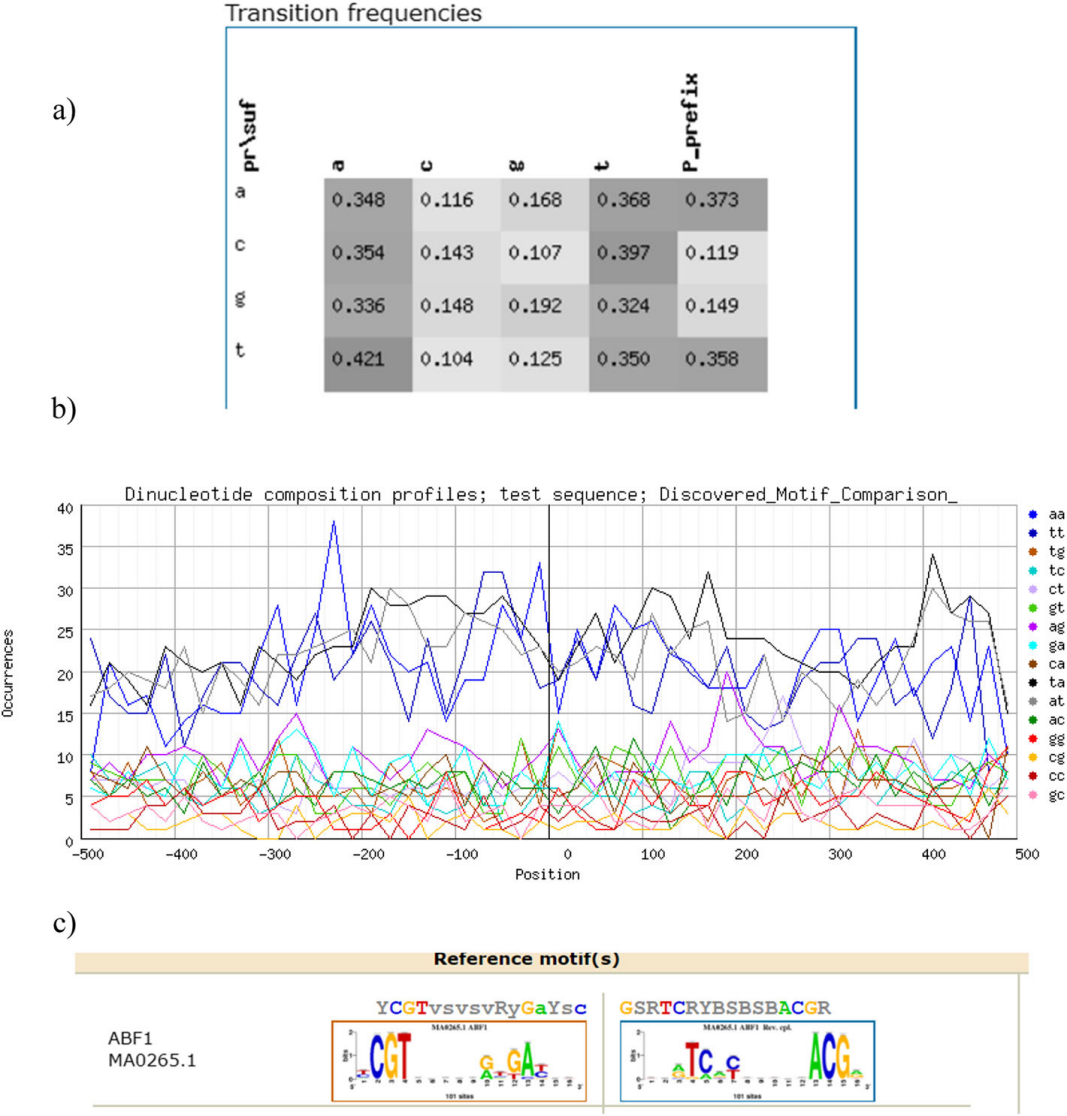
Dinucleotide composition profile (**a**), nucleotide position peaks (**b**), and number of occurrences along centered peak (**c**)

In the current study, analysis by Regulatory Sequence Analysis Tool (RSAT) resulted that the dinucleotide heat maps indicated the probability to observe a given residue with suffix and prefix displayed in columns following another residue in rows, respectively (Fig. 5a). Dinucleotide composition heat map showed typical aggregative trend of As and Ts; there was a much higher probability to observe As (42.1%) followed by minimal residue of observing C after T with 10% of proportion. Occurrence of long peak curves on the right side in between − 300 and − 200 nucleotide position and left arm position also shown long motif peaks around 400 bp peak coordinates, which were resulted possibly through integration of a serious of neighboring peaks (Fig. 5b, c).

### Determination of CpG islands

Even though in silico analysis for the 15 promoter regions and gene body regions by using Takai and Jones’ algorithm was studied, possible CpG island was not found from both regions. However, the alternative method to analyze the presence of CpG islands through in silico digestion of mitogenome *trn* gene clusters using *MspI* revealed only two fragments of CGIs for genes nad4L (110 bp) and nad5 (64 bp) at the gene body regions. Despite very few gene body regions have shown CGIs occurrence by using restriction enzyme *MspI*, the present study revealed poor possible occurrence of CGIs in both promoter and gene body regions.

### Phylogenetic analysis of mitogenome *trn* gene clusters

**Fig. 6:**
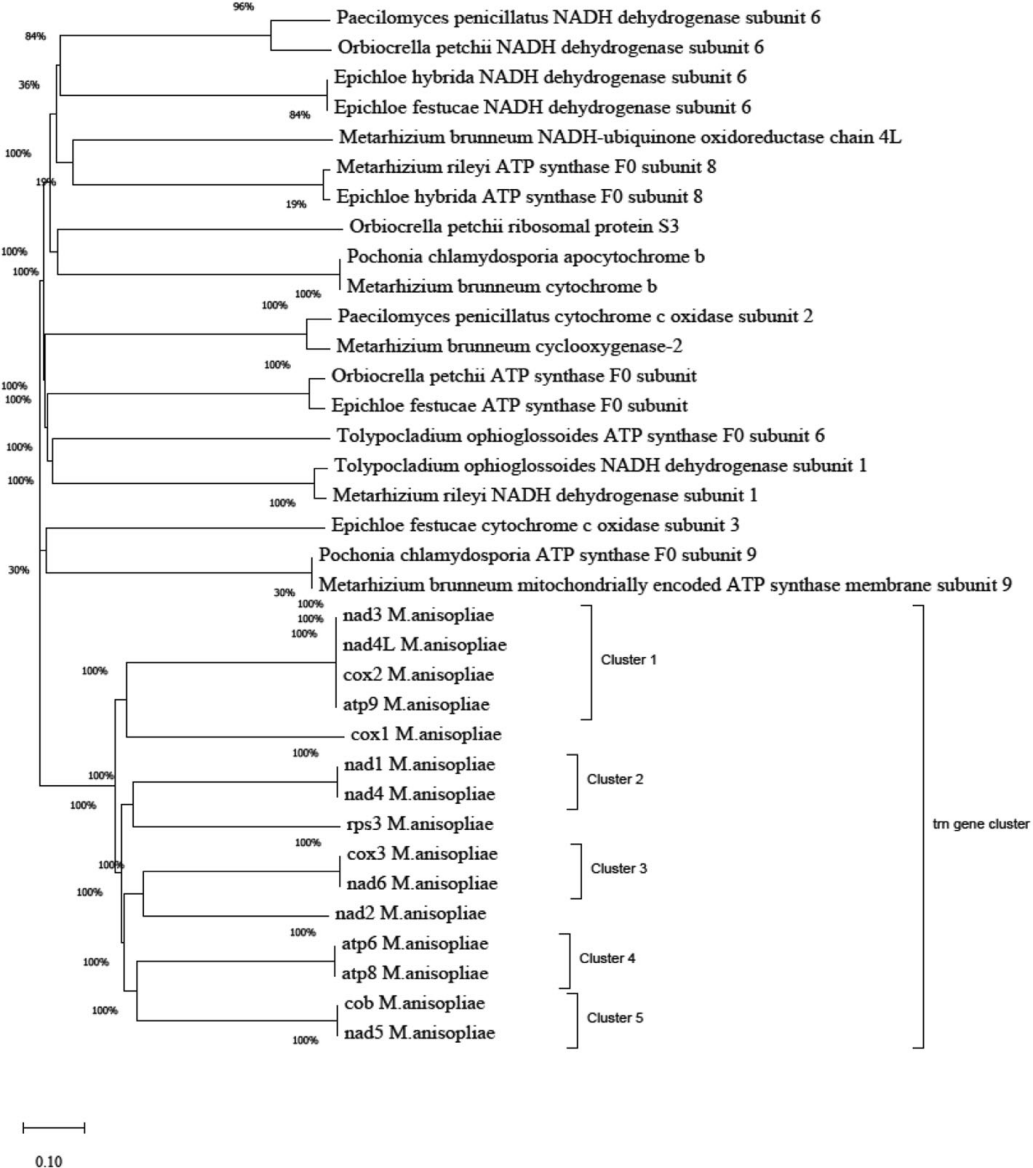
Phylogenetic tree of mitogenome *trn* gene clusters as inferred by using the Neighbor-Joining method

Phylogenetic relationship was determined for *M. anisopliae* among sequences revealed by pairwise alignment in GenBank. Genealogical relationship for the topological structures of amino acids showed that genes of *M. anisopliae* Strain ME1 were indicated ancestral to *Pochonia chlamydosporia* ATP. Accordingly, synthase F0 subunit 9 and *Metarhizium brunneum* mitochondrially encoded ATP synthase membrane subunit 9 shown ancestral relationships with a bootstrap value of 30% (Fig. 6). Based on a phylogenetic analysis inferred from amino acid sequences, *Epichloe festucae* with a gene cytochrome c oxidase subunit 3 appeared to be most common ancestral close relation to *M. anisopliae* Strain ME1 as same as *Pochonia chlamydosporia.* When comparison of gene clusters of *M. anisopliae* Strain ME1 was considered, all mitogenome *trn* gene clusters of *M. anisopliae* Strain ME1 showed homologues evolutionary ancestor supported with a bootstrap value of 100%. However, despite of identical genealogical relationship due to their shown homologues sequences, varied clustering and sister grouping was observed at the node. For example, cluster 1 contained with highest (100%) bootstrap value for nad3, nad4L, cox2, and atp9 genes, which indicates more strength and homology information.

The percentage of replicate trees in which the associated taxa clustered together in the bootstrap test (1000 replicates) was shown next to the branches. The tree was drawn to scale, with branch lengths in the same units as those of the evolutionary distances used to infer the phylogenetic tree. This analysis involved 35 amino acid sequences. All positions with less than 70% site coverage were eliminated.

## Discussion

The current study using the machine learning approach NNPP resulted in varied number of TSS, which could suggest their contribution in regulatory mechanisms. Studies reported that determining the location of TSS is very crucial and makes determination of promoters more accurate [28], since the promoter region which contains transcription factor binding sites are mostly found immediately upstream of 1 kb. Given the relationship between TSS and promoter region, the present study found multiple number of TSS (86.7%) which could likely contribute in cell-specific gene regulation roles. In agreement with this study, Dai et al. [29] discussed that genes with multiple TSS enhances the chance of transcription initiation and contribute genes in response to environmental condition changes.

In the present analysis, common motifs with different information content were identified, which might be participating in regulation of recognized promoters with contributor matching pathways. This is in agreement with Hudson and Quail [30] who reported that co-regulated genes are likely responsive to the same pathway and share common regulatory motifs. The current analysis revealed multiple binding sites in the promoter region of candidate motifs, which could be used to strengthen binding interactions and different regulatory effects. Similarly, Bilu and Barkai [31] reported that multiple occurrences of binding sites within a promoter are involved in a weaker negative selection during interactions.

As it was described by Pan et al. [32], transcription factors have a major role in fungal development, pathogenesis, and response to the environment. A common motif Mtrn I was shown as the most common regulatory motif for *M. anisopliae* Strain ME1 genes that assist as binding sites with Tryptophan cluster transcription factors, which regulate genes involved in protein fusion and in cellular metabolism. Although the machine learning approach revealed the significant matching role of Tryptophan cluster transcription factors with *Mus musculus* model, yet experimental validation whether it serve to repress or activate the gene expression of *M. anisopliae* Strain ME1 is required*.* The initiation of transcription is regulated by transcription factors [32, 33]; accordingly, this transcription factor represents a domain that patterns of a protein interact with its target site on DNA. Furthermore, shared occurrence of nuclear receptors with C4 type zinc finger in the common candidate motif could also provide some information about the possible existence of particular signaling pathways. Similarly, Li and Liu [34] described that cysteine referring C4 type zinc finger-binding domain has DNA binding biological function in basidiomycetous yeast pathogen *Cryptococcus neoformans*.

The current GO analysis observed for the common motif MtrnI with the matching database search gene CG33237 and resulted in regulatory role of protein kinase, which could play a role in gene expression and signaling pathways. Gene ontology analysis for the biological process also shown serine and threonine to regulate protein kinase; this might contribute for the activation of transcription factors and may also help to permit specific set of gene expression. The current study is in line with the work of Albataineh and Kadosh [35] who reported that responsible structural and functional genes encoding fungal catalytic protein kinase are highly conserved. The common motif MtrnI was also predicted matching with mitochondrial cytochrome b, which could have a role in mitochondrial regulation and signaling pathway in reaction of energy generation and possible roles in biosynthesis.

In silico pathway analysis for atp8 promoter region has shown a relation with only atp6 gene; this could reflect possible involvements of functional co-expression abilities. Wu and his colleagues [36] indicated that functional interaction of genes is involved in the same biochemical reactions as an input activator or inhibitor or otherwise as two members of related protein complex. Biological process sharing close interactions were detected for atp6 gene and it has shown the highest interaction with atp8 (0.49%) followed by nad4 (0.20%) and additional interaction with publically available database genes, which might be due to molecular function resulting associated level of gene expression [37].

A regulatory sequence analysis tool was employed to detect cis-regulatory elements, since RSAT tools have been used for the detection and analysis of regulatory elements [38]. Long peak curve was observed in between − 300 and − 200 nucleotide positions, which might be due to frequency of occurrence of nucleotide at the specific motif position and suggested that this region might have a strong affinity positions for binding transcription factors. Dinucleotide composition heat map showed a highest probability occurrence for A and T. This might be an indication for their abundant share in transcription initiation. In line with the current analysis, Boeva et al. [33] mentioned that AT or GC rich sequences, which are often positioned in the promoter regions, contribute in activation of transcription. Similarly, it was also indicated that a sequence composed with A/T rich motifs are serving as binding sites for the C4 type zinc finger nuclear receptor in fungi families [39]. Chen et al [40] in their study also pointed out that in *Scytalidium auriculariicola* mitochondrial genome, high frequency` of A and T use in codons contributes to the high AT content (73.70%). Therefore, promoter regions with AT-rich motifs could play a pivotal role in guiding for TSS selection.

Analysis for CpG islands on both promoter region and gene body region through enzymatic digestion resulted in poor cluster of CpG, which might be associated with selective gene expression at a specific tissue. Previous studies of CGIs of yeast by Sharif et al. [41] noticed that low CpG promoters are targeted by DNA methylation. Furthermore, Mishra et al. [42] in their study attempted that the methylated gene region in fungi are likely involved in pathogenesis or virulence. Consistent with the present analysis, previously, low G + C content was reported from fungi family Clavicipitaceae [43] and *S. auriculariicola* [40]. Despite CpG islands are clustered in unmethylated gene regions, nevertheless, gene body region CGIs densities are characterized by open chromatin and disposed to divergence so that connection to DNA damage and mutation rates are possible causes [41]. According to Teissandier and Bourc’his, concerning gene body methylation in eukaryotes, intragenic methylation levels are often associated with transcriptional strength indicating that DNA methylation is likely coupled to transcriptional elongation and consequently GC content within this region is inversely correlated [44].

Phylogenetic analysis for *M. ansiopliae* Strain ME1 genes showed homologues evolutionary ancestor based on verified 100% bootstrap value, which could be due to possible reason of predominant inheritance of mitochondrial genomes of those expected to share common genealogy. As it was previously reported by Sloan and co-workers [45], as a result of limited variation and long branch attraction, mitochondrial genes generated limited phylogenetic signals. Based on phylogenic study, rps3 gene was distinctly assigned on a separate clade instead of grouping together with other gene clusters which could be due to the presence of more introns so that results in complex evolutionary event. Consistent with the current study reports in Hypocreales group of fungi, mitogenome rps3 genes showed distinct difference among other protein coding genes [43].

## Conclusion

In this study, from the annotated mitochondrial genome sequence of *M. anisopliae* Strain ME1, promoter region and transcription regulatory elements were determined. Determination of transcription start site was a key task intended to move for identification and characterization of promoter zones, where significant regulatory elements are found playing a pivotal role in gene regulations. Accordingly, the present finding identified TSS sites and promoter sequences for 15 conserved protein coding genes of *M. anisopliae* Strain ME1. Despite varied number of TSS were identified, promoter region nad6 gene contained maximum number of TSS. This research identified common candidate motif to serve as a binding site for matching gene regulatory elements. Therefore, the overall results suggest that identified common candidate motifs and binding transcription factors through bioinformatics approach are likely expected to contribute for better understanding of gene expression of entomoathogenic fungi *M. anisopliae* Strain ME1. Based on the current finding we recommend further experimental studies to validate the role of currently identified transcription factors and their common binding sites in regulation of gene expression.

## Data Availability

The datasets used during the current study are available from the corresponding author.

## Abbreviations

Mtrn I: Motif of *trn* gene
TSS: Transcriptions start site
NNPP: Neural network promoter prediction
TFs: Transcription factors
CpG: *Cytosine-phosphate-guanine*
NCBI: National Center of Biotechnology Information
